# Assessment of Therapeutic Response to Statin Therapy in Patients With Intracranial or Extracranial Carotid Atherosclerosis by Vessel Wall MRI: A Systematic Review and Updated Meta-Analysis

**DOI:** 10.3389/fcvm.2021.742935

**Published:** 2021-10-27

**Authors:** Pengyu Zhou, Yuting Wang, Jie Sun, Yannan Yu, Mahmud Mossa-Basha, Chengcheng Zhu

**Affiliations:** ^1^Department of Radiology, Sichuan Provincial People's Hospital, University of Electronic Science and Technology of China, Chengdu, China; ^2^Department of Radiology, University of Washington, Seattle, WA, United States; ^3^Internal Medicine Department, University of Massachusetts Memorial Medical Center, Worcester, MA, United States

**Keywords:** vessel wall imaging, intracranial, carotid, atherosclerosis, plaque, statin

## Abstract

**Background and Aims:** Statin therapy is an essential component of cardiovascular preventive care. In recent years, various vessel wall MRI (VW-MRI) techniques have been used to monitor atherosclerosis progression or regression in patients with extracranial or intracranial large-artery atherosclerosis. We aimed to perform a systematic review and meta-analysis on the effects of statin therapy on plaque evolution as assessed by VW-MRI.

**Materials and Methods:** Prospective studies investigating carotid and intracranial atherosclerotic plaques in patients on statin therapy monitored by serial VW-MRI were systematically identified in the literature. The plaque burden and lipid-rich necrotic core (LRNC) volume of carotid plaque and the imaging features of intracranial plaques were extracted and summarized. For studies investigating carotid artery wall volume and LRNC volume, combined estimates were derived by meta-analysis.

**Results:** The study identified 21 studies of carotid plaque and two studies of intracranial plaque. While 16 studies investigating carotid plaques that included 780 patients by High-resolution VW-MRI were included in the meta-analysis. There was no significant change in carotid wall volume from baseline to 12 months. A significant change in LRNC volume was observed at > 12 months compared with baseline (Effect = −10.69, 95% CI = −19.11, −2.28, *P* < *0.0*1), while no significant change in LRNC volume at 3–6 months or 7–12 months after statin therapy initiation in 6 studies. Increases in fibrous tissue and calcium and reduction in neovascularization density of the plaque were seen in 2/3 studies (including 48/59 patients), 1/3 studies (including 17/54 patients), and 2/2 studies (including 71 patients) after statin therapy, respectively. Two studies with 257 patients in intracranial atherosclerosis showed that statins could effectively decrease wall volume and plaque enhancement volume.

**Conclusions:** Collective data indicated that statins could potentially stabilize carotid plaques by significantly reducing LRNC with 1 year of therapy as shown on serial carotid VW-MRI. There was no significant decrease in wall volume, which nonetheless indicated that plaque composition changes might be more sensitive to response monitoring than wall volume. It is likely that more sensitive, clinically relevant, and preferably quantitative indicators of therapeutic effects on intracranial vessel plaque morphology will be developed in the future.

## Introduction

Patients with extracranial or intracranial large-artery atherosclerosis are treated with aggressive risk factor control to lower their increased risk of ischemic stroke and other vascular events. This typically involves intensive low-density lipoprotein cholesterol (LDL-C) lowering with statin therapy. Meta-analyses of randomized clinical trials on statin therapies have shown that every 1 mmol/L reduction in LDL-C was associated with a 22% reduction in major vascular events (1). Despite LDL being a good marker for the effect of statins, residual stroke risk remains and other markers should be investigated for better pharmacology regime or monitoring the treatment effect (2).

A study in the Chinese population found that compared with the ipsilateral extracranial carotid artery, greater atherosclerotic plaque burden had a closer association with stroke severity (National Institutes of Health Stroke Scale) in the middle cerebral artery, suggesting that intracranial plaques deserve more attention in stroke etiology (3).

Vessel wall MRI (VW-MRI) is a non-Invasive imaging technique with high reproducibility and is used for characterizing atherosclerotic plaques in extracranial carotid or intracranial cerebral arteries (4–6), as well as plaque progression or regression (7–9). Studies of carotid atherosclerosis revealed the plaque characteristics shown on VW-MRI were highly consistent with those shown on histopathology (10, 11), providing the theoretical basis to evaluate the treatment response of atherosclerotic plaques by this imaging technique. Monitoring atherosclerosis with VW-MRI may enable the visual characterization of plaque morphologic and compositional evolution. VW-MRI studies have indicated that lowering LDL-C can remove the cholesterol from plaques, reduce the lipid content, induce plaque regression, even increase the plaque calcification, and therefore stabilize plaques (12–14). Moreover, monitoring atherosclerosis progression or regression in extracranial carotid or intracranial cerebral arteries with VW-MRI may allow for the identification of poor responders to standard-of-care medical therapy, who could be helped with novel lipid-lowering therapies or enrolling in clinical trials evaluating other endovascular treatments.

New studies have been published since the last meta-analysis of using VW-MRI to monitor changes of carotid plaques by Brinjikji et al. (15). The statins therapeutic response assessment in intracranial plaque by VW-MRI has not been systematically studied yet. Studies investigating novel techniques for vessel plaque evaluation are ongoing and show considerable promise. In this systematic review and updated meta-analysis, we aimed to evaluate the effect of statins on plaque components (such as lipid, fibrous tissue, and calcium) and plaque morphology (such as wall volume) as monitored by serial VW-MRI. The effects of statins in carotid plaques assessed by other advanced MRI techniques such as dynamic contrast-enhanced MRI (DCE-MRI), T2 mapping, and MRI targeting macrophages were also reviewed. The selection of parameters has been discussed. Finally, study results investigating intracranial plaques by VW-MRI after statin therapy were summarized.

## Materials and Methods

This systematic review was performed with a standardized protocol with reference to the Preferred Reporting Items for Systematic reviews and Meta-Analysis (PRISMA) guidelines. Ethical committee approval and patient consent were not required because of their statistical nature.

### Search Strategy

A comprehensive search of the PubMed, Embase, Medline, and the Cochrane library databases from January 1st, 2000 through December 31st, 2020 was performed. To identify eligible studies, the following keywords were used in combination with the Boolean operators OR and AND: “intracranial atherosclerosis” or “intracranial plaque” or “intracranial artery stenosis” or “intracranial artery disease” or “cerebral artery atherosclerosis” or “carotid atherosclerosis” or “carotid plaque” or “carotid artery stenosis” or “carotid artery disease” or “lipid-rich necrotic core” and “high-resolution magnetic resonance imaging” or “vessel wall imaging” or “plaque imaging” and “statin” or “lipid-lowering treatment” or “HMG-COA (3-hydroxy-3-methyl glutaryl coenzyme A) reductase inhibitor.” We also hand-searched references from key articles to find any additional studies on the use of serial carotid and intracranial plaque MRI to monitor response to statin therapy. Scoping searches were performed, and an iterative process was used to translate and refine the searches. The list of articles was supplemented with extensive cross-checking of reference lists within the included articles. The search was performed by a senior researcher and reviewed by a second researcher, and discrepancies in judgment were resolved by consensus.

### Eligibility Criteria and Study Selection

Specific inclusion criteria were: (1) English language articles; (2) all patients were on statin therapy or the results of the statin group were separately reported; (3) patients underwent serial vessel wall MRI to assess the response to statin therapy of intracranial and extracranial carotid atherosclerosis; (4) use of serial carotid or intracranial plaque MRI with dedicated surface coils; (5) minimum field strength of 1.5 T at all time points; (6) total sample size was > 10 patients; (7) follow-up duration was at least 3 months; (8) necessary data were reported to calculate the indicators of plaques. Attempts were made to contact the corresponding author for additional data details when necessary.

Studies must report at least one of the following regarding the plaques: (a) change in wall volume over time; (b) change in lipid-rich necrotic core (LRNC) volume over time; (c) change in calcium volume over time; (d) change in fibrous tissue volume over time.

If multiple studies were thought to contain overlapping patient cohorts and observed similar indicators, only the study with the largest sample size was retained. Review articles, conference abstracts, letters, comments, and case reports were excluded.

Exclusion criteria included retrospective and cross-Sectional studies, studies employing only imaging modalities other than vessel wall MRI, or studies in which the results of the statin arm could not be separated when multiple arms were involved, i.e., statin vs. non-Statin.

This selection strategy was prospectively chosen to provide sufficient and accurate data for a detailed systematic review.

### Methodological Quality Assessment

The methodological quality of included studies was independently assessed by two researchers using the “Guidelines for Assessing Quality in Prognostic Studies on the Basis of Framework of Potential Biases” (16). Discrepancies in judgment were resolved by consensus after discussion.

### Data Extraction and Analysis

Two reviewers independently extracted data from eligible studies, and disagreements were resolved by consensus. The following study characteristics were extracted: (1) sample size and demographics of study participants; (2) the regimen and dose of statins in all arms; (3) specific MRI protocols employed; (4) the changes in plaque imaging characteristics and in LDL-C levels at all monitoring time points.

We did the meta-analysis of studies investigating variable outcomes of carotid plaques at three time points: (a) 3–6 months, (b) 7–12 months, and (c) > 12 months following statin therapy initiation. The pooled results were expressed as an “Effect” with respective 95% CI. I^2^ calculated from Q statistic was used to examine the heterogeneity among the included studies, with I values of 0–40, 30–60, 50–90, and 75–100% representing not important, moderate, substantial, and considerable inconsistency, respectively (17). A random-effects model was applicable with an I^2^ value of over 50%, and a fixed-effects model was applicable with an I^2^ value of < 50% (18). Meta-analysis was conducted using Stata version 12 (College Station, Texas: StataCorp LP, United States).

## Results

### Study Selection and Characteristics

**Figure 1:**
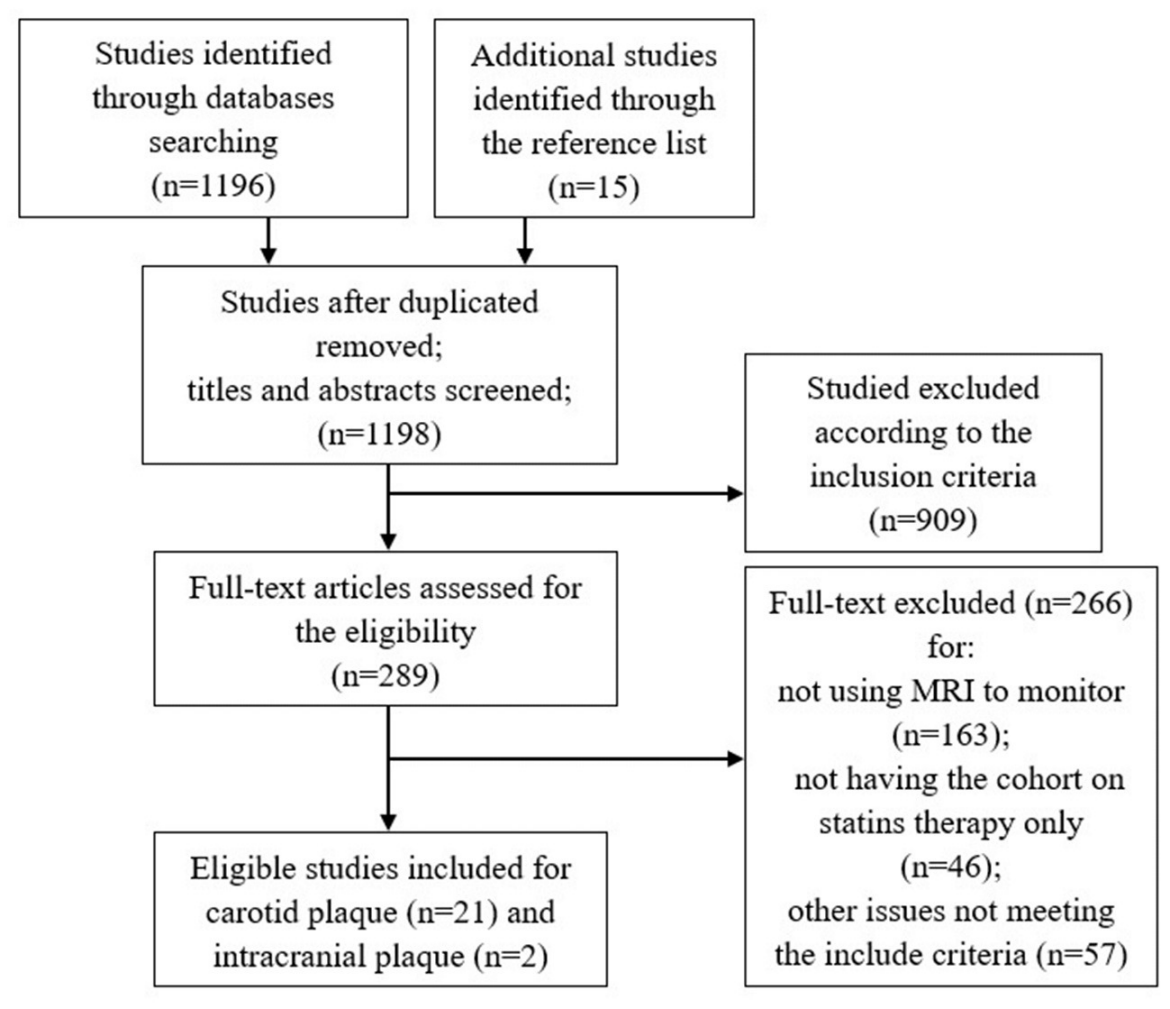
Flow diagram of literature screening and selection process.

The detailed study selection flow diagram is shown in Figure 1. A total of 1,198 articles were identified after duplicates were removed. From these, 21 articles for carotid plaque and 2 articles for intracranial plaque were identified as eligible. Limited by the number of eligible studies on the intracranial plaque, the meta-analysis included only carotid plaque vessel wall MRI studies.

Table 1 summarizes the 21 included prospective clinical studies that evaluated the changes of carotid plaques on longitudinal VW-MRI in patients on statin therapy published between 2000 and 2020. All studies evaluated the imaging characteristics of the plaques at baseline. There were 12 studies which had imaging follow up at 12 months, 4 studies at 18 months, and 9 studies at 24 months. Six studies did the measurements based on the T1 sequence. Two studies did the measurements based on the T2 sequence. One study did the measurements based on the PD (proton density) sequence. On the other hand, 12 studies did not make the corresponding statement. Eight studies drew the measurements manually, six studies used the automatic custom-designed image analysis tool (CASCADE, IBMarker Company, Seattle, WA, USA) (13, 29–31, 34, 35), and one used the semiautomatic software (Vessel Mass, Leiden University Medical Center, Leiden, Netherlands) (26). Six studies did not make the corresponding statement. All studies recorded the changes in the blood lipid index of the patients, including LDL-C and/or high-density lipoprotein-cholesterol (HDL-C). Nine studies employed plaque burden/volume as the only imaging observation at the experimental endpoint, one study employed LRNC as the only imaging observation, and seven studies used both plaque burden/volume and LRNC as observation indicators at the endpoint. In two studies investigating vascular dynamics, the dynamic changes in the parameters of the plaques were used as the only observation indicators at the endpoint of the experiment. Finally, one study employed plaque inflammation as the indicator (ultrasmall superparamagnetic iron oxide (USPIO) uptake by macrophages) and one employed distensibility as the only observation indicator at the endpoint.

**Table 1:** Serial vessel wall MRI studies on effects of statin therapy on extracranial carotid atherosclerosis.

**References**	**Research Time**	***N***	**Study design**	**Age;** **Male%**	**Medication and dose**	**Monitoring time point**	**Monitored indicators**	**Change trend of monitored indicators**	**LDL-C levels**	**Manufacturer;** **Field strength;** **Sequences**
Corti et al. (5)	2001^†^	18	Prospective, Statins only, one arm	63.5 ± 9; 56%	Simvastatin Dose unclear	Baseline, 6 months, 12 months	Plaque burden	VWA and maximal VWT decreased at 12 months and minimal VWT unchanged	159 ± 32 mg/dl at baseline; decreased by 38% at 6 weeks	GE; 1.5T; T2W
Corti et al. (6)	2002^†^	21	Prospective, Statins only, one arm	63.5 ± 9; 57%	Simvastatin Dose unclear	Baseline, 6 months, 12 months, 18 months, 24 months	Plaque burden	VWA and maximal VWT decreased at 12–24 months and minimal VWT unchanged	159 ± 32 mg/dl at baseline; decreased by 30–35% at 6 weeks	GE; 1.5T; T2W
Corti et al. (19)	1999.03–2002.03	51	Prospective, High vs. low dose statins, two arms	62.6 ± 7.6 vs 62.3 ± 10.0; NA	Simvastatin 80 vs, 20 mg/d	Baseline, 6 months, 12 months, 18 months, 24 months	Plaque burden	VWA and maximal VWT decreased at 12–24 months and minimal VWT unchanged, in both arms	173 ± 33 vs. 154 ± 31 mg/dl at baseline; 93 vs. 98 mg/dl at the first 6 weeks, then no changed, in both arms	GE; 1.5T; T2W
Boussel et al. (20)	2004.02–2007.08	89	Prospective, Detailed health questionnaire	75.4 ± 9.4; 77.5%	Statins Dose unclear	Baseline, 12 months	Plaque burden/volume	VW volume increased at 12 months	Unclear	GE; 1.5T; 2D/3D TOF; T1W
Adams et al. (21)	2004^†^	11	Prospective, Different statins or dose therapy, five arms.	69; 73%	Atorvastatin 10 or 40 mg, pravastatin 20 mg, simvastatin 20 or 40 mg	Baseline, 16 months, 24 months	Plaque burden/volume	VW volume increased at 16 and 24 months	116 ± 39 mg/dl at baseline, 92 ± 38 mg/dl at 16 months	GE; 1.5T; TOF/PDW/T1W/T2W
Underhill et al. (22)	2000.01–2004.08	33	Prospective, High vs. low dose statins, two arms	64.4 ± 2.9 vs. 66.5 ± 1.8; 70 vs. 53.9%	Rosuvastatin 40 or 80 vs. 5 mg/d	Baseline, 24 months	Plaque burden/volume, LRNC	Lumen volume, VW volume, normalized wall index, or mean wall thickness unchanged; LRNC and LRNC% decreased and fibrous tissue increased at 24 months, in both arms	145.0 ± 24.0 vs. 153.6 ± 31.0 mg/dl at baseline; decreased by 59.9 ± 3.3 vs. 38.2 ± 2.4% at 24 months	GE; 1.5T; 3D TOF; 2D PDW/T1W/T2W
Saam et al. (23)	2007^†^	68	Prospective; Detailed health questionnaire	70.3 ± 8.9; 93%	Statins Dose unclear	Baseline, 18 months	Plaque burden	Mean VWA and normalized wall index increased and mean lumen area decreased at 18 months	80.6 ± 26.0 mg/dl at baseline; unclear at end time point	GE; 1.5T; 3D TOF; PDW/T1W/T2W
Tang et al. (24)^*^	2006.07–2007.08	47	Prospective; High vs. low dose statins, two arms	70.8 ± 6.7 vs. 64.7 ± 7.6; 90 vs. 90%	Atorvastatin 80 vs. 10mg/d	Baseline, 6 weeks, 12 weeks	Inflammation	USPIO-defined Inflammation decreased in the high dose arm at 12 weeks	97.06 vs. 87.78 mg/dl at baseline; decreased by 29 vs. 1% at 12 weeks	GE; 1.5T; 2D TOF/T1W/T2W
Sadat et al. (25)^*^	2006.07–2007.08	47	Prospective; High vs. low dose statins, two arms	67 ± 2.7 vs. 72 ± 1.8; 80 vs. 75%	Atorvastatin 80 vs. 10 mg/d	Baseline, 12 weeks	Stiffness	Distensibility coefficient was higher in the high dose arm at 12 weeks	98.61 ± 10.44 vs. 101.70 ± 5.41 mg/dl at baseline; 67.29 ± 7.73 vs. 99.38 ± 8.51 mg/dl at 12 weeks	GE; 1.5T; 2D TOF/T2W
Sibley et al. (26)	2003.09–2008.12	73 vs. 72	Prospective; Statins vs. statins plus Nicotinic acid, two arms	72; 82%	Nicotinic acid 1,500 mg/d; Statins Dose unclear	Baseline, 6 months, 12 months, 18 months	Plaque burden/volume LRNC	VW volume decreased at 18 months; lumen or LRNC volume unchanged, in both arms	92.81 ± 30.94 mg/dl at baseline; 77.34 ± 19.33 mg/dl at 18 months	GE; 1.5T; T1W/T2W
Lee et al. (27)	2008^†^	24	Prospective; Statins only, one arm	66.0 ± 8.7; 83%	Simvastatin 10–20 or 40 mg/d; Atorvastatin 10 mg/d	Baseline, 3 months, 12 months	Plaque burden	Normalized vessel wall area decreased at 3–12 months	112.7 ± 38.8 mg/dl at baseline; 70.8 ± 23.1 mg/dl at 3 months. 70.8 ± 23.1 mg/dl at 12 months	Siemens; 1.5T; T2W
Lee et al. (28)	2009^†^	29	Prospective; Statins vs. statins plus nicotinic acid, two arms	65 ± 9; 94%	Nicotinic acid 2 g/d; Statins Dose unclear	Baseline, 12 months	Plaque burden	Normalized vessel wall area unchanged at 12 months, in both arms	85 ± 23 mg/dl at baseline; 64 ± 16 mg/dl at 6 months; 69 ± 21 mg/dl at 12 months	Siemens; 1.5T; T2W
Migrino et al. (29)	2011^†^	26	Prospective; Statins, three arms: increase initiation, increase, maintain	67.8 vs. 68.8 vs. 65.7; 50 vs. 85 vs. 85%	Statins Dose unclear	Baseline, 6 months	Plaque burden/volume, LRNC	Wall volume decreased in statins increasing arms (N = 13) and unchanged in statins maintain arm. LRNC and LRNC% decreased at 6 months in the total arms.	86 ± 6 vs. 96 ± 8 vs. 77 ± 9 mg/dl at baseline; 74 ± 4 vs. 73 ± 7 vs. 62 ± 6 mg/dl at 6 months	GE; 3.0T; PDW/T1W/T2W
Zhao et al. (30)	2011^†^	33	Prospective; Single therapy (included statins only), double therapy, triple therapy, seven arms	55.0 ± 8.4; 78%	Atorvastatin 10–80 mg/d; Niacin 2 g/d; Colesevelam 3.8 g/d	Baseline, 12 months, 24 months, 36 months	Plaque burden/volume, LRNC	VW volume or VW volume percentage decreased at 12–36 months; LRNC and LRNC% decreased in first 24 months; FTV decreased and FTV% increased in first 24 months; Calcium and loose matrix unchanged.	163 mg/dl at baseline; Unclear at endpoints	GE; 1.5T; 2D PDW/T1W/T2W
Du et al. (13)^**^	2009.03–2012.02	43	Prospective; Statins only, one arm	60.8 ± 9.1; 78.1%	Rosuvastatin 10–80mg/d	Baseline, 3 months, 12 months, 24 months	Plaque burden/volume, LRNC	VW volume, VW percentage and lumen volume unchanged; LRNC and LRNC% decreased in first 3 months	125.2 ± 24.4 mg/dl at baseline; 66.7 ± 17.3 mg/dl at 3 months; 65.5 ± 17.0 mg/dl at 12 months; 69.8 ± 16.6 mg/dl at 24 months	GE; 3.0T; 3D TOF; PDW/T1W/T2W
Du et al. (31)^**^	2009.03–2012.03	43	Prospective; Statins only, one arm	60.8 ± 9.1; 78.1%	Rosuvastatin 5–20 mg/d	Baseline, 3 months, 12 months, 24 months	Vascularity (V_p_), vascular permeability (K^trans^)	V_p_ decreased at 3 months; K^trans^ unchanged	130 ± 24 mg/dl at baseline; 68 ± 17 mg/dl at 3months; 68 ± 18 mg/dl at 12 months; 69 ± 7 mg/dl at 24 months	GE; 3.0T; 3D TOF; PDW/T1W/T2W
Feng et al. (32)	2013.09–2016.02	60	Prospective; High vs. low dose statins, two arms	61.3 vs. 61.0; 62.5 vs. 61.5%	Pivastatin 4 vs. 2 mg/d	Baseline, 48 weeks (12 months)	Plaque burden, LRNC	Lumen area increased; Normalized wall index, Plaque thickness and Wall area decreased; LRNC decreased. In both arms.	134.57 vs. 129.93 mg/dl at baseline; unclear at endpoints	Philips; 3.0T; 3D TOF; PDW/T1W/T2W
Alkhalil et al. (33)	2016.04–2016.11	24	Prospective; Statins only, one arm	64 ± 10; 71%	Atorvastatin 80 mg/d	Baseline, 3 months	LRNC	LRNC and LRNC% decreased; FTV unchanged; FTV% increased	109 ± 30 mg/dl at baseline; unclear at endpoints	Siemens; 3.0T; TOF/T1W/T2W
Du et al. (34)^**^	2016^†^	32	Prospective; Statins continued vs. Statins discontinued, two arms	NA; NA	Rosuvastatin; Atorvastatin; Simvastatin Dose unclear	Baseline, 24 months, 48 months	Plaque burden/volume, LRNC	VW volume percentage unchanged; LRNC and LRNC% decreased in 24–48 months; FTV, FTV% and Calcium increased in 24–48 months.	124.02 ± 26.79 vs. 125.89 ± 22.85 mg/dl at baseline; 68.19 ± 16.29 vs. 70.79 ± 17.14 mg/dl at 24 months; 93.31 ± 35.91 vs. 130.73 ± 22.08 mg/dl at 48 months.	GE; 3.0T; 3D TOF; PDW/T1W/T2W
Dong et al. (35)	2001.04–2004.05	28	Prospective; Single therapy (included statins only), double therapy, triple therapy, seven arms	55 ± 6; 82%	Atorvastatin 10–80 mg/d; Niacin 2 g/d; Colesevelam 3.8 g/d	Baseline, 12 months	Vascularity (V_p_), vascular permeability (K^trans^)	Adventitial (K ^trans^) decreased at 12 months, adventitial V_p_ unchanged.	163 mg/dl at baseline; Unclear at endpoints	GE; 1.5T; PDW/T1W/T2W
Hippe et al. (36)	2018^†^	86	Prospective; Statins vs. statins plus nicotinic acid; ezetimibe was used as needed, two arms	62; 67%	Simvastatin; nicotinic acid 1.5–2 mg/d	Baseline, 24 months	Plaque burden/volume	VW volume percentage increased in both arms.	74 vs. 72 mg/dl at baseline; 69 vs. 67 md/dl at 24 months	GE/Philips; 3.0T; NA

*Arranged by research time. N, number of patients; Male%, proportion of male; PDW, proton density weighted; T1W, T1 weighted; T2W, T2 weighted; V_p_, fractional plasma volume; K^trans^, transfer constant; VW, vessel wall; VWT, vessel wall thickness; VWA, vessel wall area; FTV, fibrous tissue volume; LRNC, lipid rich necrotic core*;

* *The same patient cohort*;

** *The same patient cohort*,

† *published time*.

Table 2 summarizes the two prospective clinical studies from 2016 to 2020 evaluating treatment response to statins based on imaging findings on longitudinal intracranial VW-MRI studies. These studies monitored plaque burden/volume, intima-media thickness, and plaque number at baseline and after 6 months of statin therapy and recorded the changes of lipid panels, such as LDL-C, HDL-C, etc. The study of Chung et al. made the measurements based on the T2 sequence manually. There was no corresponding statement in another study (4, 37).

**Table 2:** Clinical trials of monitoring the efficacy of statins therapy in intracranial atherosclerotic plaques by vessel wall imaging.

**References**	**Research Time**	***N***	**Study Design**	**Age;** **Male%**	**Medication and dose**	**Monitoring Time Point**	**Monitored Indicators**	**Change Trend of Monitored Indicators**	**LDL-levels**	**Manufacturer;** **Sequences**
Chung et al. (4)	2011.11–2017.06	77	Prospective; Statins only, one arm	62.6 ± 13.7; 61%	Atorvastatin 40–80 mg/d; Rosuvastatin 20 mg/d	Baseline, 6 months	Plaque burden/volume, Plaque enhancement volume	Wall volume, the wall area index and stenosis degree decreased but not the remodeling index; Plaque enhancement volume decreased.	125.81 ± 35.69 mg/dl at baseline; 60.95 ± 19.28 mg/dl at 6 months	Philips; 3.0T; 3D TOF; PDW/T1W/T2W
Chen et al. (37)	2016.01–2017.02	180	Prospective; Different statins, four arms	59 vs. 61 vs. 62 vs. 60; 56 vs. 60 vs. 53 vs. 51%	Four arms: Atorvastatin 20 mg/d, Simvastatin 40 mg/d, Pravastatin 20 mg/d, Rosuvastatin 10 mg/d	Baseline, 6 months	Wall volume, plaque number and intima-media thickness	Wall volume, plaque number and intima-media thickness were decreased in all arms.	Four arms, baseline vs. 3 months, mg/dl: 152.36 ± 29.00 vs. 145.78 ± 24.75; 174.01 ± 27.46 vs. 144.62 ± 37.51; 147.33 ± 37.12 vs. 150.81 ± 25.91; 159.71 ± 25.91 vs. 148.88 ± 22.43	Not mentioned clearly

*N, number of patients; Male%, proportion of male; PDW, proton density weighted; T1W, T1 weighted; T2W, T2 weighted*.

### Risk of Bias and Quality Assessment

The results of the quality assessment were generally satisfactory. There were 10 studies judged to have a low risk of bias in four assessed domains. Six studies were judged to have a moderate risk of bias in at least one domain. Common sources of moderate risk were study participation and study attrition. Study attrition bias risk came from missing data points, specifically some patients not receiving all follow-up imaging studies and/or lab values at the prescribed time points. Five studies had a high risk of bias in the domain of study participation resulting from a lack of reasonable inclusion and exclusion criteria and/or rationality in variable control. There was no study with a high risk of bias in more than one assessed domain.

### Selection of Plaque Measurements

#### Morphological Parameters

The study found that 16 studies employed wall area, wall thickness, wall area index, wall volume, and lumen volume as the morphological parameters in the clinical trials of carotid atherosclerosis (Table 1). The wall area is defined as wall area = vessel area – lumen area. The wall thickness is the mean vessel wall thickness. The wall area index is defined as the ratio of the wall area of the stenosis site to that of the reference wall. The wall volume and lumen volume are three-dimensional indicators (volume= area × slice thickness × the number of slices) (4–6, 38). The interobserver and interscan intraclass correlation coefficient (ICC) for morphological measurement reported ranged from 0.97 to 0.99. The interobserver and interscan mean coefficient of variation (CV) of the total wall volume measurements were 4.6 and 3.8%, respectively, showing good repeatability (7–9, 20–23, 33).

#### Compositional Parameters

Eight studies employed LRNC volume, fibrous tissue volume, and Calcium volume as the plaque component variables assessed on carotid VW-MRI (Table 1). The ICCs for component measurement reported in the included studies ranged from 0.88 to 0.96 (9, 22, 33).

#### Other Novel Parameters

Two studies employed parameters of vascular permeability such as transport constant (K^trans^) and fractional plasma volume (V_p_) as the endpoints of clinical medication trials. Two studies employed USPIO-defined inflammation and stiffness as novel parameters.

In atherosclerosis, macrophage accumulation initiates lesion development and triggers clinical events by elaborating various molecules that promote inflammation, plaque disruption, and subsequent thrombus formation (39–42). To identify the plaque inflammation activity, common parameters include USPIO signal intensity in the plaque and compliance coefficient (24, 25). Amax and Amin were defined as the maximum and the minimum vessel cross-sectional areas through the cardiac cycle, respectively. The distensibility was: (Amax–Amin)/Amin^*^ the average brachial pulse pressure. The compliance coefficient was: (Amax–Amin)/the average brachial pulse pressure (25).

### Response of Atherosclerosis on Statins

#### Extracranial Carotid Atherosclerosis

##### Lipid Rich Necrotic Core Volume

**Figure 2:**
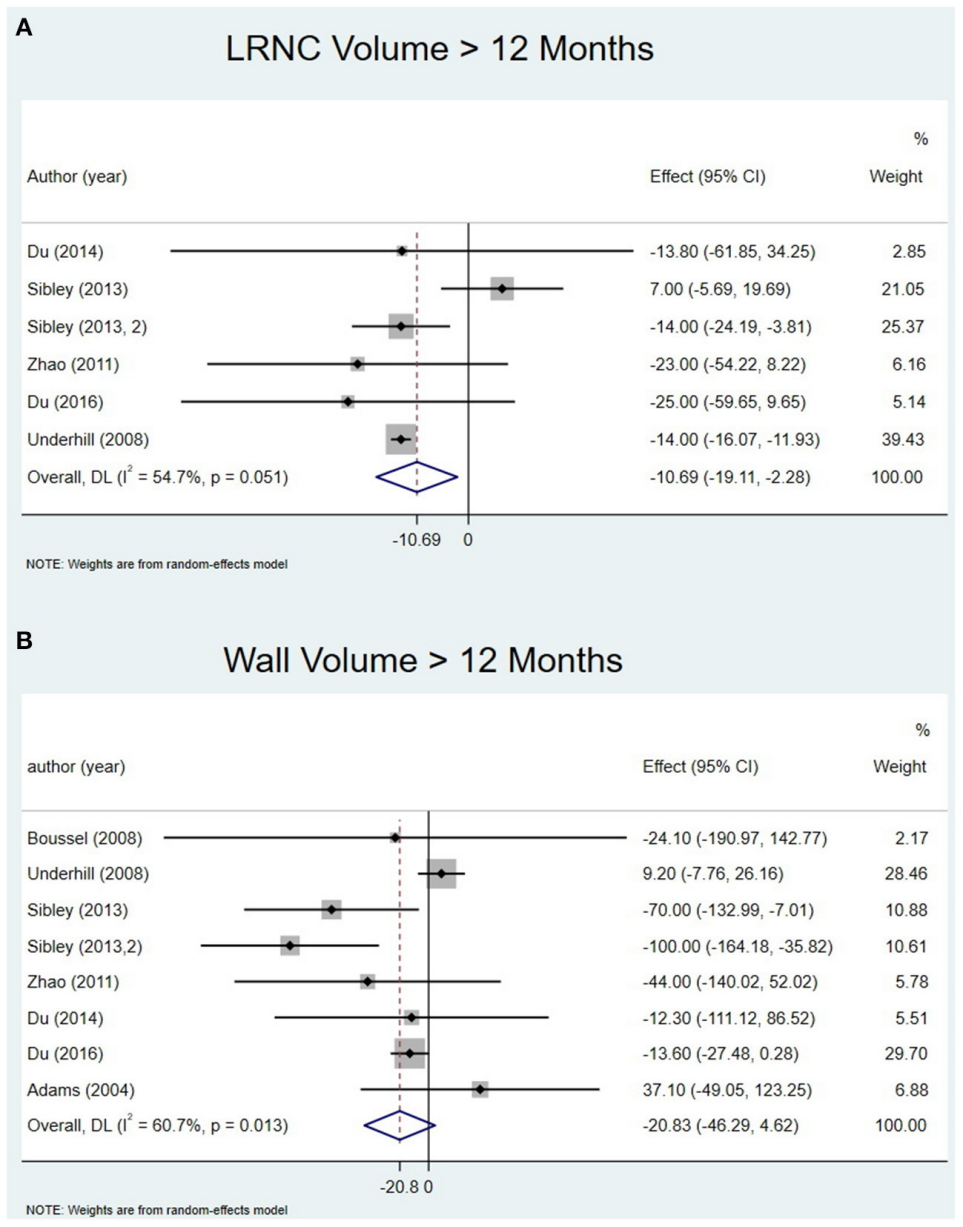
Change in LRNC volume and Wall volume at > 12 months: **(A)** forest plot of change in LRNC volume at > 12 months; **(B)** forest plot of change in Wall volume at > 12 months.

**Figure 3:**
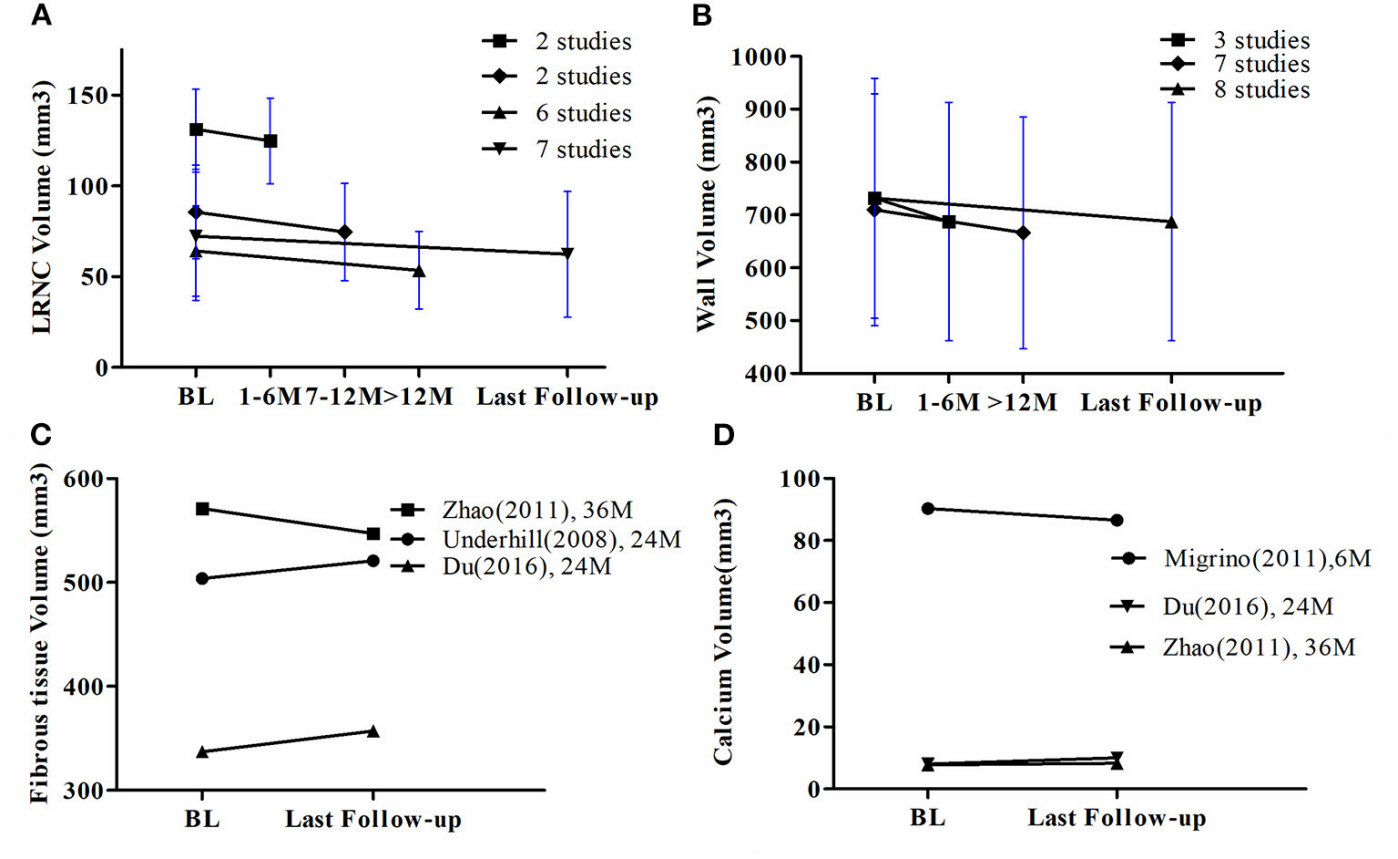
**(A)** The line chart of LRNC volume, **(B)** The line chart of wall volume, **(C)** The line chart of fibrous tissue volume, **(D)** The line chart of calcium volume. BL = Baseline. The bars mean 95% CI.

Six studies extracted the change in LRNC volume. The mean LRNC volume was 72.3 ± 35.3 mm^3^ at baseline and 62.3 ± 34.6 mm^3^ at the last follow-up. There was a significant decrease in LRNC volume at > 12 months (Effect = −10.69, 95%CI = −19.11, −2.28, *P* < 0 01). There was no significant decrease in LRNC volume at any time points < 1 year. Moderate heterogeneity was observed for the analysis of LRNC. The data is summarized in Table 3. Representative Forest plots and a detailed line chart are provided in Figures 2A, 3A.

**Table 3:** Results of meta-analysis.

	**Number of studies**	**Effect (95% CI)**	**I^2^**	**Effect (95% CI)**	**I^2^**	**Effect (95% CI)**	**I^2^**
		**3–6 months (mm3)**		**7-12 months (mm3)**		**>12 months (mm3)**	
LRNC volume	6	−5.01 (-17.65, 7.64)	0	−11.49 (-34.76, 11.77)	0	−10.69 (-19.11,−2.28)	54.7
Wall volume	8	-	-	−10.0 (-130.6, 110.6)	-	−20.83 (-46.29, 4.62)	60.7

##### Fibrous Tissue Volume

Only three studies had corresponding data and therefore meta-analysis was not performed. Two studies found an increase in fibrous tissue volume at the last follow-up. However, one study found a decreased volume at the 24-month time point after initiation of statin therapy and at the last follow-up. The detailed line chart is provided in Figure 3C.

##### Calcium Volume

Only three studies had corresponding data and therefore meta-analysis was not performed. One study found an increase in calcium volume at the last follow-up. However, two studies found a decrease and no change in calcium volume at the last follow-up, respectively. The detailed line chart is shown in Figure 3D.

##### Wall Volume

All eight studies evaluated the change in wall volume. The mean wall volume at baseline was 731.8 ± 227.1 mm^3^ and at the last follow-up was 687.2 ± 225.6 mm^3^. There was no significant decrease at > 12 months (Effect = −20.83, 95%CI = −46.29, 4.62, *P* >0.05). I-squared value was 60.7% at > 12 months indicating substantial heterogeneity. There was no significant decrease at any time point except the last follow-up. The data is summarized in Table 3. Representative Forest plots and a detailed line chart are provided in Figures 2B, 3B.

##### Neovascularization

Two studies with 71 total patients found a decrease in V_p_ or K^trans^ of plaque at 3 and 12 months after statin therapy initiation, which indicated a reduction in plaque neovascularization density.

##### The Relationship Between LRNC Volumes and LDL Levels

**Figure 4:**
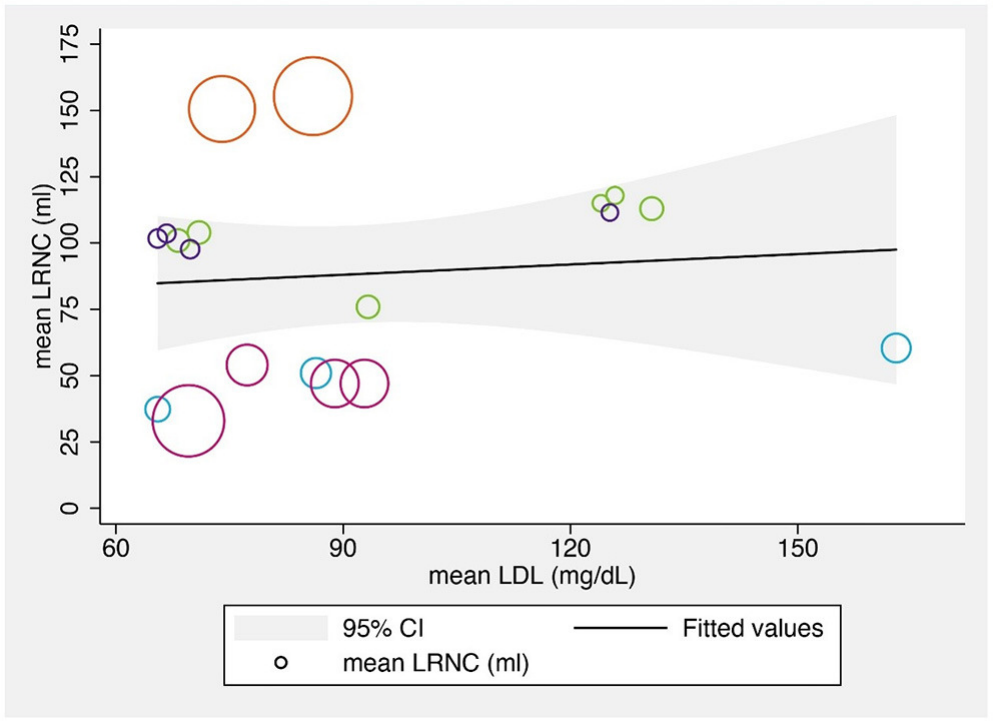
The bubble chart about the relationship between mean LRNC volumes and mean LDL-C levels.

We also explored the relationship between the mean LRNC volumes and the mean LDL-C levels at various time points. Although several previous studies found that changes in the LRNC were significantly correlated with LDL-C levels (22, 32, 36), the reported LRNC volume changes varied across the reviewed studies, possibly due to the different study populations, designs, and statin regimens. The bubble chart showing the results across included studies is shown in Figure 4.

#### Intracranial Atherosclerosis

In the evaluation of 77 patients with symptomatic acute ischemic stroke treated with high-dose statins (Atorvastatin 40–80 mg or rosuvastatin 20 mg), the study of Chung et al. found that high-dose statins could effectively decrease plaque enhancement volume, wall area index, and degree of stenosis. Longer duration (more than 1 month) of statin treatment and higher reduction of LDL-C were associated with decreased plaque enhancement volume (4, 38). Another study by Chen et al. compared the efficacy and safety of different statins in the treatment of intracranial atherosclerotic plaques involving middle cerebral arteries and found that different statins could significantly reduce blood lipid levels, intima-media thickness, plaque number, and wall volume, but rosuvastatin and atorvastatin had more effective anti-inflammatory and plaque stabilizing effects than simvastatin and pravastatin (37).

## Discussion

This updated meta-analysis of patients who received serial carotid plaque imaging while on statin therapy demonstrated a significant reduction in LRNC volume only with > 1 year of therapy, whereas wall volume did not change significantly at any time points. It suggested that VW-MRI could visualize the changes of plaque components and morphology *in vivo* after statin therapies, and the former could be a more sensitive marker of therapeutic response. Increasing LRNC size was associated with the development of new ulceration, fibrous cap rupture, increasing plaque burden, as well as ischemic stroke events (43–46). Therefore, LRNC size was a good target for the evaluation of therapeutic response. A review of studies that monitored changes of intracranial atherosclerotic plaques by VW-MRI suggested that changes in multiple parameters such as decreased plaque number, wall volume, and plaque enhancement volume could be readily detected.

Compared with the meta-analysis by Brinjikji et al. (15) which also explored the change of plaque characteristics after statin therapies, our analysis provided additional information listed below. (1) One new prospective study with both vessel wall and LRNC data were included (34); (2) we summarized the change in plaque components such as fibrous tissue and calcium in addition to LRNC volume and wall volume; (3) the relationship between mean LRNC volumes and mean LDL-C levels were plotted; (4) given the technical developments in plaque imaging, a review of advanced MRI techniques including T2 mapping and DCE was performed and the established indicators for therapy monitoring of atherosclerotic plaques were summarized; (5) the results derived from studies of intracranial atherosclerosis were also summarized.

### Carotid Atherosclerosis

Two of three studies showed a significant increase in fibrous tissue volume of carotid plaques after 2 years of statin treatment. The timing of the change suggested that the decrease in the LRNC volume probably preceded the increase in fibrous tissue volume, which could be consistent with the proposed mechanism that the LRNC component might be replaced by fibrous tissue after statin treatment (15).

Calcium volume did not change significantly at any time points including for follow-up duration as long as 3 years (30). Sun et al. (44) reported that high plaque calcification content was not associated with poor prognosis in AIM-HIGH. Calcium might not be the crucial component to evaluate in carotid plaques when a therapeutic response of statins is being assessed by VW-MRI.

The study of Hellings et al. (47) found that carotid plaque features including IPH and intraplaque vessel formation identified in endarterectomy specimens were associated with future cardiovascular events in 818 patients who underwent carotid endarterectomy. Currently, advanced MRI techniques such as DCE MRI and USPIO enhanced MRI show unique advantages in characterizing plaque micro-vascularization and inflammation. These parameters could be further explored in future prospective studies to monitor the effects of therapy on plaque morphology (48–51).

### Intracranial Atherosclerosis

Because of the unique structure and environment, intracranial arteries receive nutrient supply directly from cerebrospinal fluid and have fewer vasa vasorum than carotid arteries (52). Fewer vasa vasorum, however, makes intracranial arteries more vulnerable to hypoxic injury. The hypoxic environment caused by pathological wall thickening in atherosclerosis can stimulate angiogenic factors (e.g., vascular endothelial growth factor) that consequently promote neovascularization (53–55). Statins can reduce the release of vascular endothelial growth factors through anti-inflammatory effects, thereby reducing neovascularization and plaque enhancement volume on MRI seen in carotid plaque (35, 56). These observations suggest that statins might have different mechanisms to stabilize intracranial plaques than extracranial ones, and the assessment of intracranial plaque changes requires further investigation, which is indeed necessary given the crucial role that intracranial atherosclerosis plays in stroke development. Notably, a recent study on intracranial atherosclerosis indicated that 35% of patients had a poor response to statin therapy; post-Treatment VW-MRI showed no change or increased plaque enhancement volume and increased degree of stenosis. The study suggested that these patients might need to choose alternative therapeutic options such as PCSK9 inhibitors or a combination with ezetimibe to lower lipid levels (4).

Regarding the selection of measurements on imaging, several clinical studies provide a theoretical basis and report practical experience for the assessment of responses to statin therapy in extracranial atherosclerosis, however, the clinical studies investigating intracranial atherosclerotic plaques are relatively few, with a limited assessment of VW-MRI characteristics. The consensus of experts of the American Society of Neuroradiology on intracranial vessel wall imaging along with a recent meta-analysis indicates that intracranial plaque enhancement, positive remodeling, and plaque irregularity are associated with increased risk of stroke (57, 58). These features are therefore highly relevant when assessing and validating response to therapy. Furthermore, evaluation of plaque compositional characteristics in addition to morphological features on VW-MRI might be clinically relevant as suggested by the imaging features of carotid plaques. Given the unique role of neovascularization in the pathogenesis of vessel plaques, other novel techniques to assess neovascularization should be developed.

### Imaging Techniques

The standard multi-contrast black blood MRI (fast-spin-echo based) for carotid vessel wall imaging has been widely used for the evaluation of plaque morphology, and the sequences are widely available on the major commercial scanners at both 1.5 and 3T. All included studies used specific neck surface coils to improve the image signal-to-noise ratio/resolution. However, the neck surface coils are not widely available in clinical radiology departments and a separate purchase is required. Without using neck surface coils, the clinical head-neck or neurovascular coils produce noisier images which limit the image resolution and therefore limit the measurement accuracy of plaque morphology. This is a major barrier for clinical translation and multi-center trials.

In the past decade, 3D fast-spin-echo sequences with variable flip angle train (product sequence in all major vendors, termed SPACE in Siemens, CUBE in GE, and VISTA in Philips) have been widely used for carotid vessel wall imaging due to its high scan efficiency, isotropic resolution, and intrinsic blood suppression (59, 60). Such 3D techniques allow high-resolution imaging using clinical neurovascular coils (61), which significantly improve their feasibility in a clinical setting.

While the evaluation of plaque volume and LRNC volume using standard multi-contrast black blood MRI is the most popular and feasible approach, more advanced but less popular methods including T2 mapping, DCE and USPIO-MRI have their unique advantages. The T2 mapping method provides quantitative analysis of the plaque component and is more reproducible across scanners and centers. The DCE method provides unique information on the vasa vasorum, which is an important factor in plaque vulnerability. USPIO-MRI provides unique information of the vessel function and inflammation (macrophage activity). However, their disadvantages are also apparent. For the T2 mapping and DCE techniques, specific sequences are required, and specific post-Processing is required, too. Thus, only a few research centers performed such analysis. The USPIO contrast is off-label for medical imaging usage. Thus, despite that its safety profile is comparable to gadolinium, it is not widely available for imaging, and only a few centers performed USPIO MRI (62). In addition, to visualize inflammation, two imaging sessions (1–3 days apart) are required to enable macrophages to uptake the contrast agent. There is still a big gap to fill before these novel techniques can be used more widely. Better sequences and easier post-Processing pipelines will improve the availability of these techniques in the future.

## Conclusion

Collective data indicated that statin therapy could stabilize carotid plaques by significantly reducing LRNC of therapy as shown on serial carotid VW-MRI. Future studies and clinical practice could focus on changes in plaque characteristics (such as components) rather than morphological features. It is expected that more sensitive, clinically relevant, and preferably quantitative indicators to monitor therapeutic effects on intracranial plaques will be developed. VW-MRI may play an important role in assessing and guiding clinical statin therapies in the future.

## Data Availability Statement

The original contributions presented in the study are included in the article/Supplementary Material, further inquiries can be directed to the corresponding authors.

## Author Contributions

PZ, YW, and CZ contributed to conception and design of the study. PZ organized the database, performed the statistical analysis, and wrote the first draft of the manuscript. YW, CZ, JS, MM-B, and YY wrote sections of the manuscript. All authors contributed to manuscript revision, read, and approved the submitted version.

## Funding

This work was supported by the Research Project of Healthcare for Cadres of Sichuan province, Grant No. 2021-229. CZ is supported by United States National Institute of Health (NIH) grant R00HL136883.

## Conflict of Interest

The authors declare that the research was conducted in the absence of any commercial or financial relationships that could be construed as a potential conflict of interest.

## Publisher's Note

All claims expressed in this article are solely those of the authors and do not necessarily represent those of their affiliated organizations, or those of the publisher, the editors and the reviewers. Any product that may be evaluated in this article, or claim that may be made by its manufacturer, is not guaranteed or endorsed by the publisher.
